# Extensive left and right ventricular metastasis from myxoid liposarcoma

**DOI:** 10.1093/ehjcr/ytae183

**Published:** 2024-04-09

**Authors:** Meriem Boumaaz, Sara Ahchouch, Raid Faraj, Iliyasse Asfalou

**Affiliations:** Department of Cardiology, Mohammed V Military Hospital, Mohammed V University in Rabat, Rabat, Morocco; Department of Cardiology, Mohammed V Military Hospital, Mohammed V University in Rabat, Rabat, Morocco; Department of Cardiology, Mohammed V Military Hospital, Mohammed V University in Rabat, Rabat, Morocco; Department of Cardiology, Mohammed V Military Hospital, Mohammed V University in Rabat, Rabat, Morocco

## Case description

**Figure 1:**
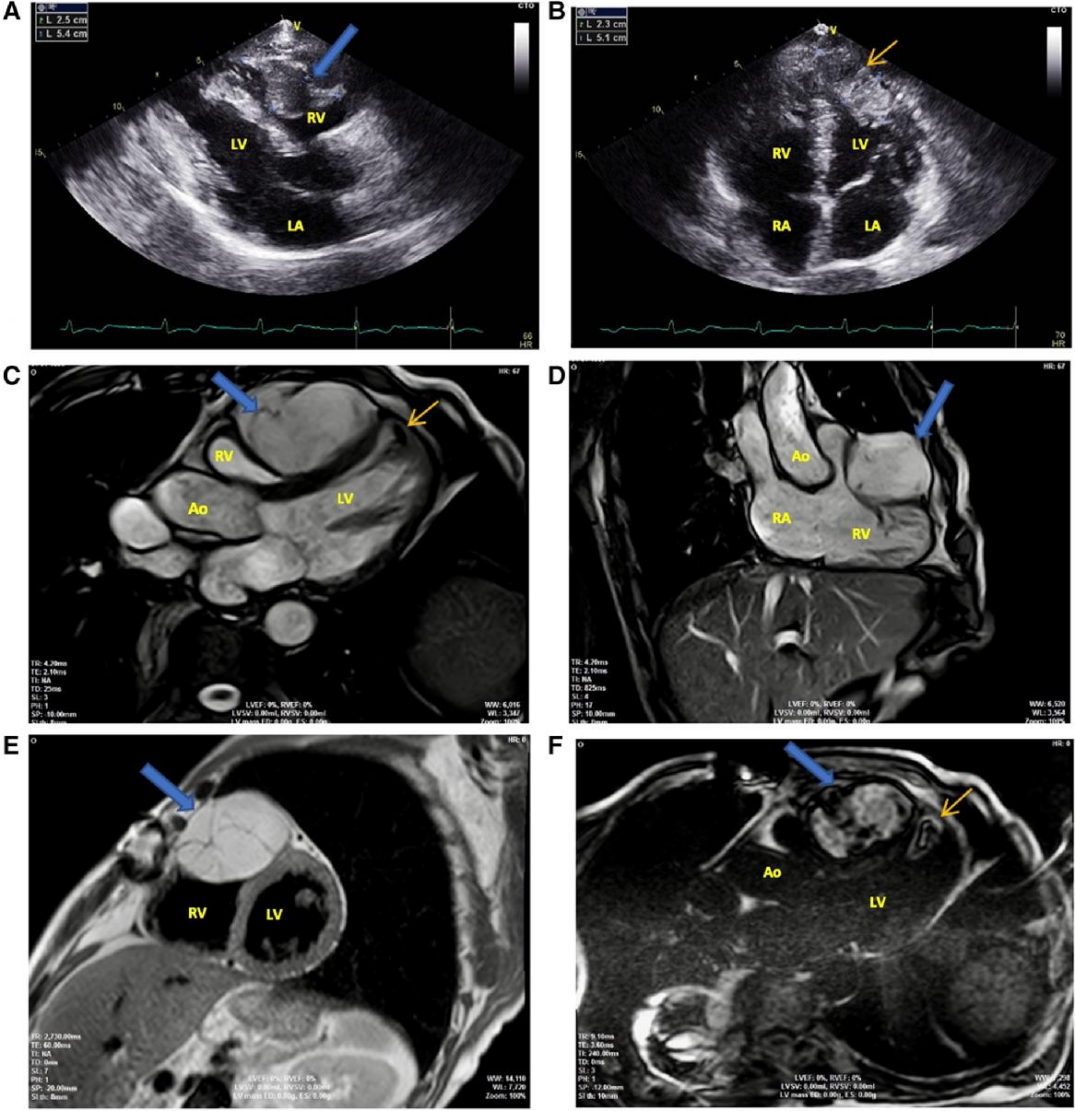
Transthoracic echocardiogram (TTE) and cardiac magnetic resonance imaging (CMR) findings. (*A*) TTE parasternal long-axis view revealing an oblong, non-vascularized intra-myocardial mass attached to the right ventricle (RV) free wall’s myocardium (blue arrow). (*B*) TTE apical view revealing an oblong non-vascularized intra-myocardial mass in the antero-septal-apical wall of the left ventricle (LV), extending to the antero-lateral-apical wall (orange arrow). (*C*) CMR three-chamber view showing an intra-myocardial mass on the anterior wall of the RV (blue arrow) with compression effect associated with a small mass on the antero-septal-apical wall of the LV (orange arrow). (*D*) CMR RV outflow view showing an intra-myocardial mass on the anterior wall of the RV (blue arrow). (*E*) Fat–water separation sequences in short-axis view characterizing the non-adipose nature of the RV mass which appears in higher signal intensity with the presence of septations. (*F*) Late gadolinium enhancement (LGE) sequences in three-chamber view showing a heterogeneous signal of the two cardiac masses.

A 74-year-old male with a past medical history significant for myxoid liposarcoma (MLS) of the right thigh, which was treated with wide excision associated with adjuvant chemotherapy and radiotherapy, presents 3 years after the surgery for the primary tumour, a follow-up chest computed tomography showing two low-density areas in both the right (RV) and left ventricles (LV). The patient was then asymptomatic. Transthoracic echocardiogram (TTE) identified an oblong non-vascularized intra-myocardial mass, measuring 23 × 51 mm, in the antero-septal-apical wall of the LV, extending to the antero-lateral-apical wall (*Figure 1B*, orange arrow). The other rounded mass was discovered attached to the myocardium of the RV free wall and protruding into the cavity (*Figure 1A*, blue arrow) measuring 54 × 25 mm, which was suggestive of a neoplasm. Clinical images revealed no evidence of local recurrence or distant metastasis other than the cardiac masses at that time. Cardiac magnetic resonance imaging (CMR) confirmed these findings and showed a rounded formation embedded in the apical segment of the antero-septal myocardial LV wall, measuring 30 × 19 mm with regular outline (*Figure 1C*, orange arrow), and a second rounded formation with a partitioned structure in the anterior wall of the RV, embedded in the myocardial wall with regular contour and measuring 49 × 23 mm (*Figure 1C* and *D*, orange arrows). These formations have a homogeneous structure: isosignal intensity on T1-weighted images; higher signal intensity on T2-weighted images and higher signal intensity on T2 fatsat with the presence of septations characteristic of MLS due to its high water content (*Figure 1E*, blue arrow), with no early contrast at first perfusion sequence; and heterogeneous enhancement on late gadolinium enhancement sequences due to its variable vascularity and fibrous components (*Figure 1F*, orange and blue arrows). Considering the clinical course, these masses were regarded as metastasis of MLS. The patient refused surgical excision and was therefore treated by radiotherapy. During the follow-up, the patient remained asymptomatic, with the masses observed in the CMR control showing no significant change after 1 year.

Metastatic cardiac liposarcoma is extremely rare; however, follow-up including examination for cardiac lesions is necessary long after resection of the primary lesion.

## Data Availability

Data sharing is not applicable to this article as no data sets were generated or analysed during the current study.

## Lead author biography

Dr Meriem BOUMAAZ is a cardiologist in the cardiology department of Mohammed V Military Hospital, Mohammed V University in Rabat - Morocco. She was a cardiologist in the cardiovascular Functional Explorations department and in the Nuclear cardiology department at the East hospital Group, Louis Pradel Hospital of Lyon University Hospital in France. She had also worked as a cardiologist specializing in cardiac imaging in the department of Radiology, diagnostic imaging and interventional, at the Nord hospital, Saint-Étienne University Hospital in France. Her research activities focus on cardiac imaging MRI and cardiac CT, on coronary artery disease and thrombosis, on cardiomyopathies and on valvular heart disease. She has authored numerous papers in qualified scientific journals. Dr Boumaaz is a Member of the Moroccan Society of Cardiology and the Moroccan Association of Cardiology. And through her work she participates in various national and international cardiology scientific events.

**Consent:** The authors confirm that written consent for the submission and publication of this case, including images, has been obtained from the patient and his parents in line with the Committee on Publication Ethics (COPE) guidance.

**Funding:** None declared.

